# Dietary and body composition profiles in Chinese pediatric late-onset Pompe disease: implications for nutritional management

**DOI:** 10.3389/fmed.2026.1795679

**Published:** 2026-04-02

**Authors:** Xinting Liu, Yong Zhang, Xueyuan Guo, Linyan Hu, Peili Hu, Gang Zhu, Xinyun Yao, Jun Li, Guang Yang

**Affiliations:** 1 Senior Department of Pediatrics, Chinese PLA General Hospital, Beijing, China; 2 Department of Pediatrics, First Medical Center, Chinese PLA General Hospital, Beijing, China; 3 Medical School of Chinese People's Liberation Army, Beijing, China; 4 Department of Nutrition, the First Medical Center of Chinese PLA General Hospital, Beijing, China; 5 Department of Rehabilitation Medicine, the First Medical Center of Chinese PLA General Hospital, Beijing, China; 6 The School of Medicine, Nankai University, Tianjin, China

**Keywords:** body composition, dietary intake, gastrointestinal symptoms, late-onset Pompe disease, six-minute walk test

## Abstract

**Background:**

Late-onset Pompe disease (LOPD) is characterized by glycogen accumulation, leading to progressive weakness and gastrointestinal involvement. This study aimed to investigate the dietary habits and body composition phenotype of pediatric patients and to assess their correlations with motor ability and gastrointestinal symptom burden.

**Methods:**

In this study, body composition was assessed by bio-electrical impedance analysis (BIA), and dietary intake was quantified using a food-frequency questionnaire. Clinical outcomes comprised the Gastrointestinal Symptom Rating Scale (GSRS) and the 6-min walk test (6MWT). Associations between variables were examined by correlation analyses.

**Results:**

This study enrolled 30 children with LOPD and 30 healthy controls. Compared with controls, patients with LOPD exhibited significantly lower body mass index (BMI), intracellular water (ICW), total body water (TBW), skeletal muscle mass (SMM), protein mass (PM), and basal metabolic rate (BMR) (all *P* < 0.05). Children with LOPD displayed a markedly lower daily frequency of fruit intake, and increased consumption of dairy products and eggs. Correlation analyses revealed that multiple body composition parameters (ICW, SMM, etc.) were positively associated with motor performance (*P* < 0.05). For diet, soy product and dairy intakes correlated positively with 6-min walk distance (*r* = 0.389, *P* = 0.045; *r* = 0.386, *P* = 0.048), while vegetable intake correlated negatively with gastrointestinal symptom severity (*r* = −0.366, *P* = 0.047).

**Conclusion:**

LOPD children exhibited pronounced alterations in body composition and dietary pattern. These findings support the integration of nutritional assessment and dietary intervention into the comprehensive management of pediatric LOPD.

## 1 Introduction

Pompe disease (PD) is an autosomal recessive disorder characterized by lysosomal glycogen accumulation caused by a deficiency of acid α-glucosidase (GAA) (1). The principal sites of glycogen storage include skeletal muscle, heart, liver, and smooth muscle, and the resulting clinical manifestations are largely determined by age at onset (2, 3). Infantile-onset Pompe disease (IOPD), which presents in infancy, is fatal and leads to severe cardiac and respiratory failure (4, 5). Late-onset Pompe disease (LOPD), which typically presents in adolescence or adulthood, generally follows a slowly progressive course, predominantly causing proximal lower limb weakness and respiratory insufficiency (6, 7).

Enzyme replacement therapy (ERT) with alglucosidase alfa was approved in China in 2016 and has markedly improved cardiac function and survival (8). However, long-term survivors now manifest novel clinical symptoms, including metabolic derangements, systemic vasculopathy, and involvement of gastrointestinal smooth muscle (9). Children with LOPD face unique challenges: those in the adolescent period are undergoing critical growth and development, and their disease management differs substantially from that of adults. Myopathic dysfunction can precipitate gastrointestinal symptoms and feeding difficulties, impairing nutritional intake and body composition and thereby accelerating disease progression, affecting growth and development, and quality of life (10).

Current guidelines already recommend dietary intervention as an adjunct to ERT in LOPD to preserve muscle function and limit glycogen accumulation (2, 11). Bioelectrical impedance analysis (BIA) offers a rapid, non-invasive means to quantify skeletal muscle mass, adipose tissue, and hydration status (12). Although these body composition parameters are critical for motor performance in neuromuscular disorders, their relationships with dietary intake and functional outcomes in children with LOPD remain incompletely defined.

This study aimed to describe the distinctive feeding practices and dietary intake of children with LOPD, together with their early-phase body composition profile, and the impact of dietary patterns and body composition variables on motor ability and gastrointestinal burden, thereby guiding dietary modifications to achieve improved clinical outcomes in the LOPD population.

## 2 Materials and methods

### 2.1 Study design and population

This study was conducted at the Department of Pediatrics, the First Medical Center of Chinese PLA General Hospital, from February 2025 to December 2025. Patients diagnosed with LOPD were recruited from the outpatient clinic. The inclusion criteria were as follows: (i) Fulfilled the diagnostic criteria for LOPD, including progressive proximal myopathy with/without respiratory compromise, elevated creatine kinase, reduced GAA enzyme activity, biallelic pathogenic GAA variants detected by next-generation sequencing, and supportive muscle histopathology showing glycogen storage and vacuolar changes. Associated systemic features included macroglossia, hepatomegaly, and spinal deformities (13, 14); (ii) Age 6–18 years, both sexes; (iii) Signed informed assent/consent. Exclusion criteria included: (i) Inability to stand or walk without assistance. Healthy controls without neuromuscular disorders, obesity, or malnutrition were enrolled and matched for age and sex.

### 2.2 Measurements

#### 2.2.1 Anthropometric measurements

All anthropometric indices were obtained using standardized procedures. Weight and height were measured with an ultrasonic stadiometer (HW-900Y). Height was determined by non-contact ultrasound (range 20–200 cm, graduation 0.1 cm), and weight by a load cell (range 5–200 kg, graduation 0.1 kg). Participants were asked to stand upright on the platform wearing light clothing without shoes. Body mass index (BMI) was calculated as weight in kilograms divided by the square of height in meters (15).

#### 2.2.2 Bioelectrical impedance analysis (BIA)

Whole body BIA was performed with a Body Composition Analyzer (BCA-C; Beijing Sihai Huachen Technology Co., Ltd, China) using a constant high frequency current source (50 kHz, 500 μA) and an eight-point tactile electrode system in a four-polar configuration to assess body composition.

Participants were asked to stand barefoot on the electrode platform with feet parallel and knees fully extended, while gripping the hand electrodes to ensure continuous contact (16). A low intensity alternating current (below 90 μA) was passed through the body and impedance was recorded over a range of 75.0–1,500.0 Ω (17). Measured parameters included body mass index (BMI), intracellular water (ICW), extracellular water (ECW), total body water (TBW), skeletal muscle mass (SMM), fat free mass (FFM), protein mass (PM), mineral mass (MM), body fat mass (BFM), basal metabolic rate (BMR), waist-to-hip ratio, and body fat percentage (BF%).

Whole body impedance measurements were performed in the morning following an overnight fast of at least 8 h and abstinence from food and sugar-containing or caffeinated beverages. Prior to measurement, the participant's sex, date of birth, and height were entered into the Body Composition Analyzer software.

#### 2.2.3 Dietary assessment

Foods listed in the questionnaire were grouped into eight categories according to the revised Chinese Diet Balance Index (DBI-2022): cereals, soy products, vegetables, fruits, dairy, red meat and poultry, fish and shrimp, and eggs (18). Each participant was asked to recall their food intake over the past 3 months, specifying the frequency and number of servings of each item they typically consumed. Frequency options were classified into the following level: rarely, once per month, 2–3 times per month, once per week, 2–3 times per week, 3–4 times per week, 4–5 times per week, 5–6 times per week, once per day, twice per day, and three or more times per day. Standard portion sizes were defined as one egg, 250 ml of milk, and a fist-sized amount (≈ adult hand) for cereals, vegetables, and meat, etc. Responses were converted to daily intake, for example, “2–3 times per week” was coded as 0.36 portions per day. To further evaluate the differences between actual daily food intake and recommended dietary intake, as well as protein intake status in LOPD patients, we calculated the percent daily reference intake (%DRI) as (actual daily intake / recommended daily intake) × 100 based on the Chinese Food Guide Pagoda (2022 Edition) (19–21), and calculated the protein content of each food and total daily protein intake based on the Chinese Food Composition Table (22). We then compared both %DRI and protein intake between LOPD patients and healthy controls.

### 2.3 Six-minute walk test (6MWT)

The 6MWT was performed along a flat, level corridor. Participants were instructed to cover as much distance as possible within 6 min. Heart rate, SpO_2_, and Borg dyspnoea score were obtained pre- and post-test. The actual walking distance was measured and expressed as a percentage of the predicted value. All procedures followed the American Thoracic Society guidelines (23).

### 2.4 Gastrointestinal symptom rating scale (GSRS)

The 15-item GSRS was self-completed by participants, with each item scored on a 7-point Likert scale of discomfort: 1 = none, 2 = minor, 3 = mild, 4 = moderate, 5 = moderately severe, 6 = severe, and 7 = very severe. The items aggregate into five symptom domains: abdominal pain, reflux syndrome, diarrhea syndrome, indigestion syndrome, and constipation syndrome (24, 25).

### 2.5 Statistical analysis

Statistical analyses were performed with IBM SPSS Statistics 27.0. Normally distributed variables are presented as mean ± SD, non-normally distributed variables as median (interquartile range, IQR), and categorical data as n (%). Group differences between children with LOPD and controls in general characteristics, dietary habits, and body composition were tested by an independent samples *t*-test for parametric variables and Mann–Whitney U test for non-parametric variables. Categorical variables were compared with the χ^2^ test. Correlations were evaluated using Pearson or Spearman coefficients. A two-sided *P* < 0.05 was considered statistically significant. Sample size was validated through *post-hoc* power analysis using G^*^Power 3.1 software (two-sided α=0.05, power=80%).

## 3 Results

### 3.1 Participants' characteristics

A total of 30 children with LOPD and 30 age and sex matched healthy controls were enrolled. Baseline demographic characteristics are presented in Table 1. There were no significant differences in sex distribution or age between LOPD patients and controls. Although the median height and weight were lower in the LOPD group compared to controls, these differences did not reach statistical significance. Six LOPD patients had never received ERT, whereas the remaining 24 were receiving recombinant human acid α-glucosidase (rhGAA) infusions either regularly or irregularly. The 30 children with LOPD had a mean disease duration of 4.67 ± 3.42 years. LOPD patients reported significantly more severe gastrointestinal symptoms than the control group, with median (IQR) scores of 21.00 (9.75) versus 16.50 (6.00), *P* = 0.023. In particular, diarrhea and indigestion symptom scores were markedly higher than in healthy controls.

**Table 1:** Comparison of demographic characteristics and GSRS scores between the LOPD and control groups.

Characteristics	LOPD patients	Controls	*P* value
Age (years)	11.53 ± 3.98	11.43 ± 4.78	0.93
Sex, *n* (%)			1
Boys	13 (43.33%)	13 (43.33%)	
Girls	17 (56.67%)	17 (56.67%)	
Weight (kg)	29.50 (23.95)	40.20 (24.47)	0.487
Height (m)	1.47 (0.38)	1.52 (0.31)	0.114
GSRS scores	21.00 (9.75)	16.50 (6.00)	0.023^*^
Abdominal pain	3.00 (2.00)	3.00 (2.00)	0.465
Reflux syndrome	2.00 (0.00)	2.00 (0.00)	0.053
Diarrhea syndrome	5.00 (4.25)	3.00 (1.00)	0.001^**^
Indigestion syndrome	5.00 (3.25)	4.00 (1.25)	0.044^*^
Constipation syndrome	4.00 (2.25)	3.00 (1.25)	0.235

The data are expressed as mean ± standard deviation, median (interquartile range, IQR), or proportions. The statistical analyses utilized the independent samples t-test, the chi-square test, and the Mann–Whitney U test. GSRS, gastrointestinal symptom rating scale.

^*^P value < 0.05

^**^P value < 0.01

### 3.2 Comparison of body composition between LOPD patients and controls

**Figure 1:**
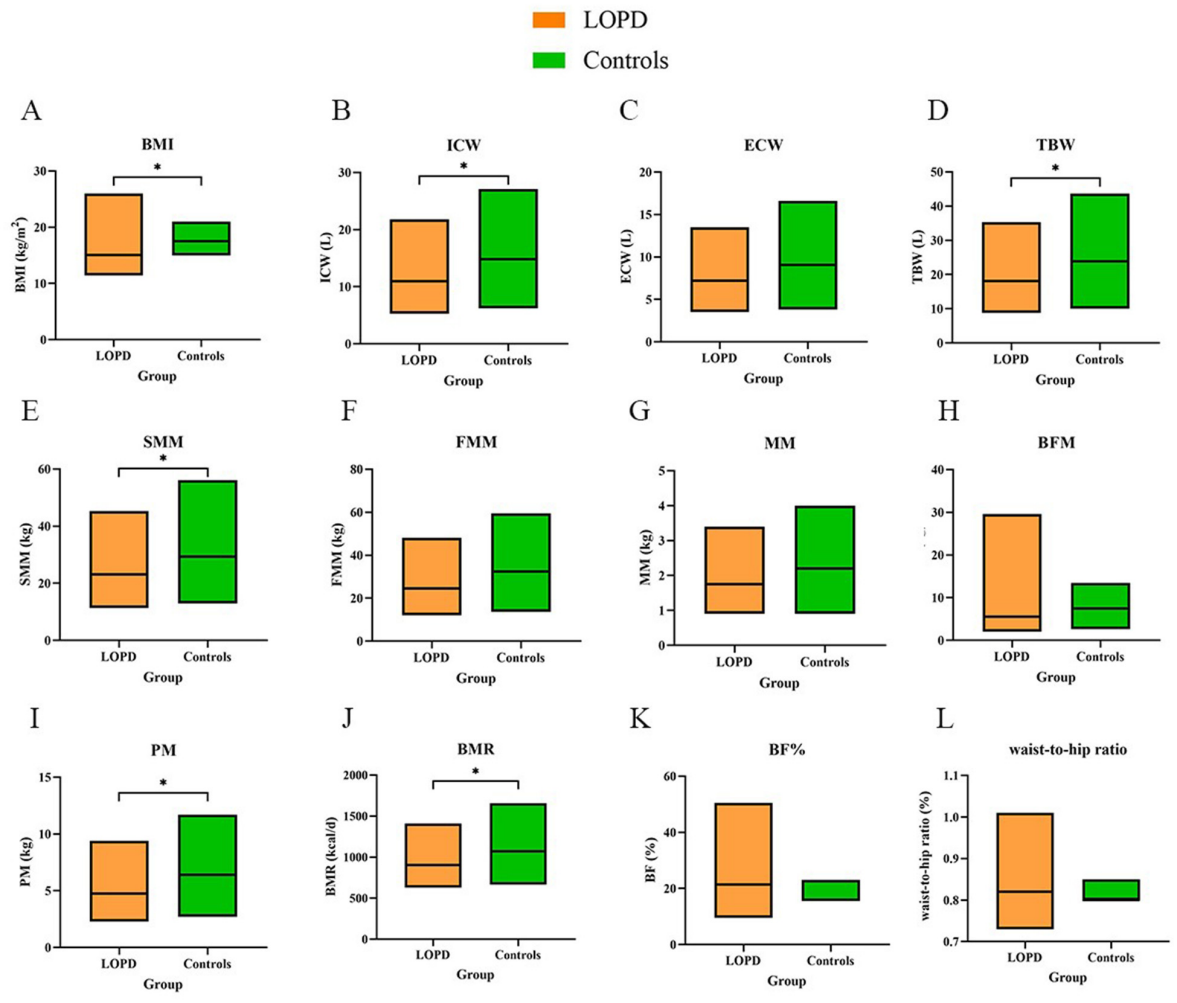
Differences in BIA parameters between the LOPD and controls. **(A)** BMI, body mass index; **(B)** ICW, intracellular water; **(C)** ECW, extracellular water; **(D)** TBW, total body water; **(E)** SMM, skeletal muscle mass; **(F)** FFM, fat free mass; **(G)** MM, mineral mass; **(H)** BFM, body fat mass; **(I)** PM, protein mass; **(J)** BMR, basal metabolic rate; **(K)** BF%, body fat percentage; **(L)** waist-to-ratio. *P* value < 0.05^*^. Box plots are presented as median (minimum, maximum).

Patients with LOPD exhibited significantly lower BMI, ICW, TBW, SMM, PM and BMR (*P* < 0.05). Parameters related to adiposity, including FFM, MM, BFM, BMR, waist-to-hip ratio, and BF%, did not differ between the two groups (*P* > 0.05) (Figure 1). Based on the BMI growth references and curves for Chinese children aged 0–18 years (26), 12 (12/30, 40.00%) children with LOPD had a BMI ≤ the 3rd percentile (P_3_), and only one child (1/30, 3.33%) had a BMI > the 95th percentile (P_95_). Whole body bioelectrical impedance analysis revealed pronounced differences in body composition between children with LOPD and controls.

### 3.3 Differences in dietary habits between children with LOPD and healthy peers

**Figure 2:**
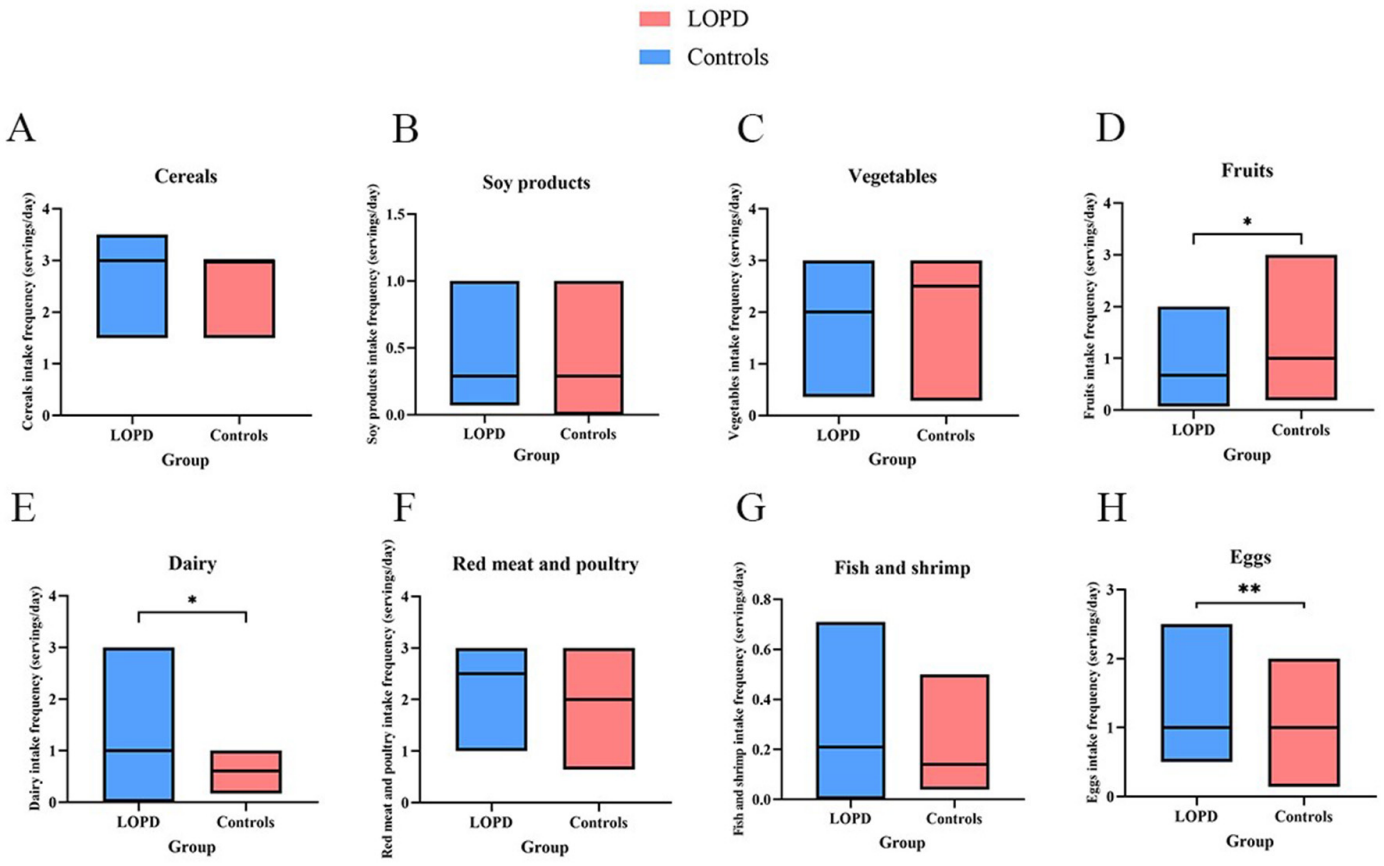
Food intake frequency/servings in LOPD and controls. **(A)** cereals; **(B)** Soy products; **(C)** Vegetables; **(D)** Fruits; **(E)** Diary; **(F)** Red meat and poultry; **(G)** Fish and shrimp; **(H)** Eggs. ^*^ *P* value < 0.05. *P* value < 0.01^**^. Box plots are presented as median (minimum, maximum).

**Figure 3:**
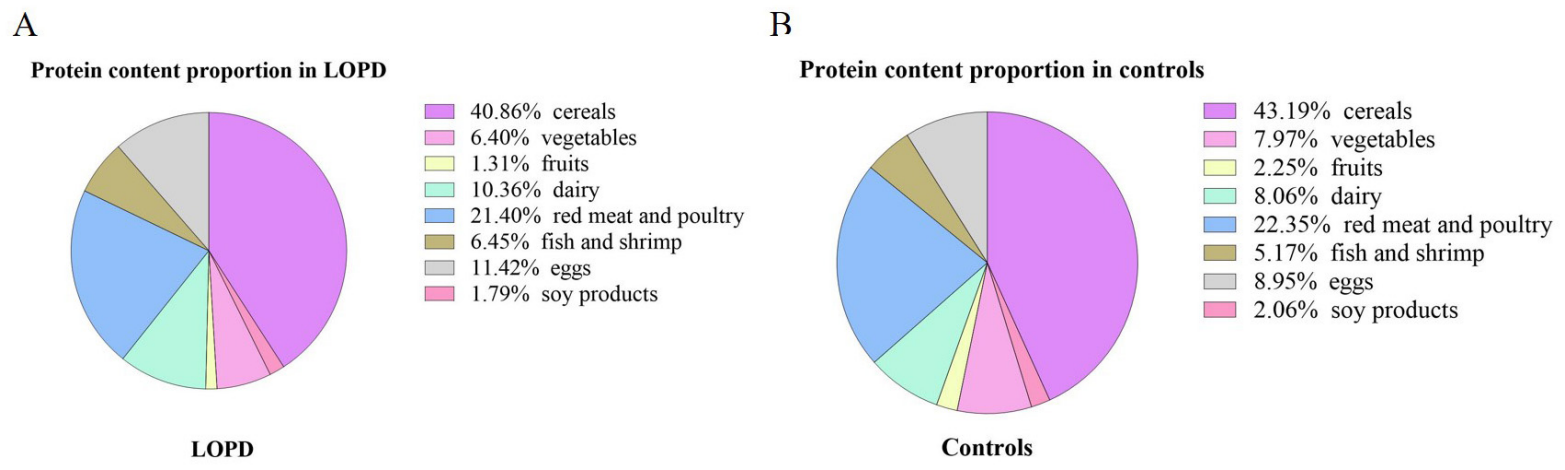
The proportion of protein provided by each food category, including cereals, soy products, vegetables, fruits, dairy, red meat and poultry, fish and shrimp, as well as eggs.

Figure 2 illustrates the daily servings of each kind of food in LOPD patients relative to matched healthy controls. Compared with controls, children with LOPD exhibited a distinct dietary pattern: daily servings of fruit intake were significantly lower (*P* < 0.05), whereas consumption of dairy products and eggs was significantly increased (*P* < 0.05). No significant differences were observed for cereals, soy products, vegetables, fish/shrimp, or red meat and poultry (all *P* > 0.05). Compared with the control group, LOPD patients had a lower %DRI for fruits, while the %DRI for eggs was higher (*P* = 0.016, *P* = 0.004). However, the median intake of soy products, dairy products, and fish and shrimp was less than 1 in both patients and controls, suggesting that none of these foods met the daily recommended intake (Table 2). LOPD patients had a median daily protein intake of 70.28 (17.62), which was higher than that of the control group [64.21 (17.95), *P* = 0.022]; cereals, red meat and poultry were the main sources of protein (Figure 3).

**Table 2:** Comparison of the percent daily reference intake (%DRI) between children with LOPD and controls.

Food category	LOPD patients	Controls	*P* value
Cereals	1.00 (0.00)	1.00 (0.17)	0.161
Soy products	0.29 (0.31)	0.29 (0.31)	0.988
Vegetables	1.00 (0.81)	1.25 (0.56)	0.221
Fruits	0.68 (0.66)	1.00 (0.43)	0.016^*^
Diary	0.63 (0.58)	0.38 (0.34)	0.173
Red meat and poultry	2.50 (1.00)	2.00 (0.63)	0.222
Fish and shrimp	0.72 (0.82)	0.48 (0.78)	0.120
Eggs	1.00 (0.50)	1.00 (0.42)	0.004^**^

^*^P value < 0.05.

^**^P value < 0.01.

The data are expressed as median (interquartile range, IQR).

### 3.4 Correlations between diet and body composition with motor outcomes in LOPD children

To investigate the correlation between dietary habits and body composition with exercise ability in children with LOPD, the 6MWT was used to evaluate the exercise ability of LOPD patients. Spearman analysis showed that multiple BIA-derived indicators were significantly and positively correlated with 6MWT performance, including ICW (*r* = 0.415), ECW (*r* = 0.406), TBW (*r* = 0.413), SMM (*r* = 0.411), FFM (*r* = 0.406), PM (*r* = 0.408), MM (*r* = 0.392), and BMR (*r* = 0.407) (all *P* < 0.05) (Table 3). Among dietary factors, soy product (*r* = 0.389, *P* = 0.045) and dairy (*r* = 0.386, *P* = 0.047) intake frequencies showed significant positive associations with 6MWT outcomes (Table 4).

**Table 3:** The correlations between BIA parameters and 6MWT in LOPD patients.

BIA parameters	*r*	*P*
BMI (kg/m2)	0.040	0.843
ICW (L)	0.415	0.031^*^
ECW (L)	0.406	0.036^*^
TBW (L)	0.413	0.032^*^
SMM (kg)	0.411	0.033^*^
FFM (kg)	0.406	0.035^*^
PM (kg)	0.408	0.035^*^
MM (kg)	0.392	0.043^*^
BFM (kg)	−0.003	0.987
BMR (kcal/d)	0.407	0.035^*^
Waist–to–hip ratio	0.000	1.00
BF%	−0.196	0.327

6MWT, six-minute walk test; BIA, bioelectrical impedance analysis; BMI, body mass index; ICW, intracellular water; ECW, extracellular water; TBW, total body water; SMM, skeletal muscle mass; FFM, fat free mass; PM, protein mass; MM, mineral mass; BFM, body fat mass; BMR, basal metabolic rate; BF%, body fat percentage.

r-correlation coefficient.

^*^P value < 0.05.

**Table 4:** The correlations between frequency/servings of dietary variables and 6MWT in LOPD patients.

Food intake frequency/servings	*r*	*P*
Cereals	0.049	0.808
Soy products	0.389	0.045^*^
Vegetables	0.212	0.289
Fruits	0.089	0.658
Dairy	0.386	0.047^*^
Red meat and poultry	0.075	0.709
Fish and shrimp	−0.017	0.934
Eggs	−0.317	0.107

6MWT, six-minute walk test.

r-correlation coefficient.

^*^P value < 0.05.

### 3.5 Dietary habits, body composition, and gastrointestinal function in LOPD Children

Tables 5, 6 present the correlations between the intake frequency of each food group and body composition variables with gastrointestinal symptoms in patients with LOPD. Among all food categories, only vegetable intake showed a significant inverse correlation with the total GSRS score as well as with the Abdominal pain and Indigestion syndrome subscales (*r* = −0.366, *P* = 0.047; *r* = −0.384, *P* = 0.036; *r* = −0.363, *P* = 0.049), indicating that higher vegetable consumption was associated with milder gastrointestinal symptoms. In contrast, the intake frequency of red meat and poultry exhibited a positive correlation with constipation syndrome. None of the body-composition parameters showed a significant correlation with the total GSRS score; however, they were inversely associated with the severity of constipation.

**Table 5:** Association between body composition and GSRS scores in LOPD patients.

Variables	Statistical indicator	BMI (kg/m^2^)	ICW (L)	ECW (L)	TBW (L)	SMM (kg)	FFM (kg)	PM (kg)	MM (kg)	BFM (kg)	BMR (kcal/d)	BF%	waist-to-ratio
GSRS scores	*r*	−0.421	−0.005	0.000	0.005	−0.006	0.006	0.001	0.037	−0.197	0.008	−0.287	0.049
	*P*	0.021^*^	0.978	1.000	0.981	0.977	0.977	0.995	0.847	0.296	0.968	0.131	0.796
Abdominal pain	*r*	−0.123	0.038	0.032	0.042	0.032	0.032	0.037	0.045	−0.121	0.042	−0.185	0.040
	*P*	0.517	0.843	0.868	0.824	0.867	0.865	0.846	0.814	0.525	0.825	0.336	0.833
Reflux syndrome	*r*	−0.080	0.157	0.139	0.152	0.117	0.129	0.162	0.133	0.106	0.143	−0.037	0.221
	*P*	0.674	0.408	0.463	0.422	0.539	0.497	0.392	0.485	0.578	0.450	0.851	0.240
Diarrhea syndrome	*r*	−0.456	−0.105	−0.097	−0.093	−0.096	−0.082	−0.101	−0.065	−0.230	−0.090	−0.270	−0.060
	*P*	0.011^*^	0.579	0.610	0.624	0.615	0.666	0.596	0.733	0.222	0.635	0.157	0.752
Indigestion syndrome	*r*	−0.118	0.261	0.253	0.264	0.256	0.253	0.266	0.270	0.050	0.266	−0.124	0.329
	*P*	0.536	0.164	0.178	0.158	0.172	0.177	0.156	0.149	0.792	0.156	0.521	0.076
Constipation syndrome	*r*	−0.465	−0.403	−0.383	−0.290	−0.392	−0.382	−0.399	−0.350	−0.374	−0.386	−0.273	−0.287
	*P*	0.010^**^	0.027^*^	0.037^*^	0.033^*^	0.032^*^	0.037^*^	0.029^*^	0.058	0.041^*^	0.035^*^	0.152	0.124

GSRS, gastrointestinal symptom rating scale; BMI, body mass index; ICW, intracellular water; ECW, extracellular water; TBW, total body water; SMM, skeletal muscle mass; FFM, fat free mass; PM, protein mass; MM, mineral mass; BFM, body fat mass; BMR, basal metabolic rate; BF%, body fat percentage.

r-correlation coefficient.

^*^P value < 0.05.

^**^P value < 0.01.

**Table 6:** Association between food intake frequency and GSRS scores in LOPD patients.

Variables	Statistical indicator	Cereals	Soy products	Vegetables	Fruits	Diary	Red meat and poultry	Fish and shrimp	Eggs
GSRS scores	*r*	−0.88	−0.018	−0.366	−0.151	0.008	0.150	0.017	0.305
	*P*	0.643	−0.925	0.047^*^	0.425	0.965	0.430	0.927	0.101
Abdominal pain	*r*	−0.69	−0.017	−0.384	−0.115	−0.033	−0.035	−0.123	0.219
	*P*	0.718	0.931	0.036^*^	0.546	0.864	0.856	0.516	0.246
Reflux syndrome	*r*	−0.181	0.168	−0.201	−0.202	−0.360	−0.168	−0.276	0.320
	*P*	0.339	0.376	0.287	0.284	0.051	0.374	0.141	0.084
Diarrhea syndrome	*r*	−0.066	0.016	−0.329	−0.033	0.248	0.073	0.064	0.135
	*P*	0.730	0.933	0.076	0.863	0.187	0.701	0.738	0.478
Indigestion syndrome	*r*	−0.010	0.013	−0.363	−0.193	−0.080	0.067	−0.208	0.193
	*P*	0.957	0.945	0.049^*^	0.308	0.675	0.725	0.269	0.306
Constipation syndrome	*r*	−0.056	−0.184	−0.291	−0.239	−0.039	0.417	0.081	0.210
	*P*	0.769	0.330	0.119	0.203	0.837	0.022^*^	0.670	0.265

GSRS, gastrointestinal symptom rating scale.

r-correlation coefficient.

^*^P value < 0.05.

## 4 Discussion

This study investigated the nutritional status, body composition, and dietary patterns of Chinese children with LOPD and evaluated their associations with clinical outcomes. Compared with controls, LOPD patients exhibited distinctive food preferences and significant alterations in body composition and metabolism. These nutritional indices were meaningfully related to gastrointestinal and motor function.

BIA revealed pronounced alterations in LOPD: BMI, ICW, TBW, SMM, PM and BMR were all significantly lower than in controls, whereas FFM, MM, BFM, BF% and waist-to-hip ratio did not differ. These findings indicate that cellular hydration, muscle mass, protein reserves and global metabolic status are compromised in children with LOPD, while adipose tissue and mineral content remain preserved. The decline in BMI without a concurrent change in waist-to-hip ratio suggests that muscle and protein loss predominantly affects the extremities rather than the trunk. Although axial and/or proximal muscle weakness is the classic phenotype of Pompe disease (27), axial muscle involvement can lead to severe complications such as scoliosis and respiratory insufficiency. Consequently, early disease stages are likely dominated by loss and atrophy of limb muscles, a notion indirectly corroborated by muscle MRI findings (28). The pattern of reduced muscle mass without significant adipose change observed in pediatric LOPD differs from that in adult LOPD, where low skeletal muscle mass is also ubiquitous but is accompanied by decreased FFM and increased BFM. Ravaglia S et al. (29) reported that among 17 adult patients with LOPD, five (5/17, 29.41 %) exhibited an increase in fat mass (FM). Papadimas GK et al. (30) used dual-energy X-ray absorptiometry (DXA) to find that eight of nine adult patients were obese based on adiposity (>30% body fat), and three individuals exceeded 50% body fat. Despite marked muscle loss, children with LOPD maintain a high metabolic rate driven by growth, which suppresses adipose accumulation. In adulthood, the lower basal metabolic rate, together with disrupted muscle metabolic pathways and myocellular steatosis, predisposes to sarcopenic obesity (29, 31). We also observed a significant reduction in cellular hydration among children with Pompe disease. In contrast, a 15-year longitudinal study of 15 adults with LOPD documented altered FM/FFM ratios in three patients, yet normal hydration status was preserved in all participants (32). This discrepancy likely reflects the accumulation of large

volumes of intracellular water within skeletal muscle fibers during growth to meet elevated metabolic demands; when these fibers are damaged or intra- versus extracellular osmotic gradients change, intracellular water leaks out, producing measurable alterations in hydration status.

We documented distinct dietary preferences among children with LOPD and found that, compared with healthy peers, they consumed significantly fewer servings of fruit but more dairy and eggs, whereas their intakes of cereals, soy products, fish/shrimp, vegetables, red meat, and poultry were comparable. This dietary pattern may represent parents' adaptive choices for disease management. The Juvenile and Adult Pompe Disease Management Guidelines recommend a high-protein diet supplying 20–25 % of total energy from protein, 30–35 % from carbohydrate, and 35–40 % from fat, with special attention to vitamins and minerals, and changing food consistency (33). Diets rich in protein and/or branched-chain amino acids and relatively low in carbohydrates have demonstrated beneficial effects on muscle function and metabolic control (34, 35). A dietary survey of 74 US. children with PD aged 1–6 years revealed that 39 (52.70 %) followed a regimen discouraging sugar items (candy, desserts, sweetened beverages, fruit juice, etc.) and processed foods, 10 (13.51 %) were encouraged to consume protein rich foods, and 18 (24.32 %) had no dietary restrictions (36). In this study, patients were also strictly restricted from consuming high sugar foods (e.g., fruits) and were provided with increased quantities of protein rich foods such as dairy products and eggs, while no limitations were placed on cereal intake. The findings indicate that the majority of Chinese Pompe disease families are aware of and adhere to dietary recommendations aimed at increasing protein intake. However, %DRI results showed that the intake of soy products, dairy products, and fish/shrimp failed to meet the daily recommended dietary intake. Soy products and fish/shrimp provided the highest amount of protein per 100g, at 25g and 18g, respectively, yet the proportion of daily protein derived from these two food sources was remarkably low. Increasing the consumption of soy products and fish/shrimp represents an easily overlooked issue among parents of Chinese patients with Pompe disease.

Correlation analyses between food intake frequency and distance of 6MWT revealed a positive association between soy product and dairy consumption and motor performance. Notably, exercise performance correlated with hydration status, protein mass, mineral content and fat free mass, whereas no significant relationship was observed with fat mass. These findings provide a more precise perspective on the factors governing motor capacity in LOPD patients, demonstrating that not only specific food categories but also adequate systemic hydration and protein reserves are critical for maintaining motor function in this population. LOPD patients who receive high protein and low carbohydrate diets have a slower decline in muscle function (37–39), but our study did not find any impact on exercise ability from carbohydrate rich foods such as grains and fruits. Previous work suggests that dairy-derived proteins exert a more potent effect than plant sources such as soy (40, 41). The superiority of whey protein over plant-based proteins lies in its more balanced essential amino acid profile, particularly its high leucine content, which effectively stimulates muscle protein synthesis via mTOR activation (34). Parents also exhibited a marked preference for providing their children with LOPD supplementary protein in the form of dairy products and eggs in our study. The contribution of hydration status to motor performance should not be overlooked. Muscle function is highly water-dependent. Adequate intracellular water and overall systemic hydration are fundamental for maintaining the efficiency of muscle contraction and ensuring the delivery of nutrients (42). Glycogen deposition within muscle fibers disrupts energy metabolism in late-onset Pompe disease and may create a dehydration risk (43), thereby influencing prognosis independently of dietary intake. Future studies should incorporate biochemical indices of hydration (e.g., serum osmolality, urine specific gravity) to refine the assessment of water balance. Although previous work has emphasized the superiority of whey over plant proteins, our results indicate that soy-derived proteins also contribute to muscle function recovery, which may be overlooked by parents. Soy protein may interact synergistically with hydration status and protein mass. Despite its lower leucine content, plant-derived protein can still facilitate muscle repair, possibly via mTOR or other pathways such as enhanced insulin-like growth factor-1 secretion (44, 45). Collectively, these data underscore the need to emphasize combined plant- plus animal-protein supplementation during the management of pediatric Pompe disease, while simultaneously monitoring hydration status and individualizing protein mass assessment in clinical practice.

Furthermore, we observed that increasing vegetable intake was beneficial for alleviating gastrointestinal symptoms, particularly abdominal pain and indigestion. This highlights the advantageous role of fiber-rich foods in improving gastrointestinal function (46).

This study has several limitations. Its cross-sectional design precludes causal inferences, and dietary intake data were self-reported, introducing potential recall bias. In future studies, a continuous 7-day or 24-h dietary record combined with a weighing method could be employed further to improve the accuracy of short-term dietary intake data. It is necessary to conduct larger, multicenter intervention trials to determine whether optimized dietary intake can increase muscle mass, alleviate gastrointestinal symptoms, and improve functional outcomes in children with LOPD. In addition, integrative analyses of the gut microbiota and metabolomics may provide deeper insights into the mechanisms and efficacy of nutritional interventions.

## 5 Conclusion

This study evaluated dietary patterns, body composition, and their clinical correlates in Chinese children with LOPD, offering insights for nutritional management. Compared with healthy controls, pediatric patients exhibited pronounced reductions in hydration status, BMR, SMM, and PM, whereas no excess adiposity was detected. It is distinct from adult LOPD and likely attributable to the interaction between catabolism and the high metabolic demands of growth. We further identified a specific dietary pattern in LOPD patients characterized by lower fruit but higher dairy and egg consumption, reflecting the adaptive nutrition strategies adopted by most families to comply with recommended high-protein regimens intended to preserve muscle function. SMM, ICW, and intakes of soy products and dairy were positively associated with 6-min walk distance, while vegetable consumption was inversely related to gastrointestinal symptom severity, providing targets for guiding functional prognosis improvement. Integrating body composition and dietary assessment underscores the importance of comprehensive care in rare diseases and supports a paradigm shift from ERT alone to an “ERT + nutritional support” model.

## Data Availability

The original contributions presented in the study are included in the article/supplementary material, further inquiries can be directed to the corresponding author.
